# Ultrastructural characterization of tau pathology in the human post‐mortem Alzheimer’s disease brain

**DOI:** 10.1002/alz.094941

**Published:** 2025-01-09

**Authors:** Daniel A. Stähli, Louis Travers, Eglantine Vialaneix, Philippine L. Schneider, Wilma D.J. Van de Berg, Henning Stahlberg, Amanda J. Lewis

**Affiliations:** ^1^ EPFL, Lausanne, Vaud Switzerland; ^2^ Amsterdam Neuroscience, brain imaging, Amsterdam Netherlands; ^3^ Amsterdam Neuroscience, Neurodegeneration, Amsterdam Netherlands; ^4^ Amsterdam UMC ‐ location VUmc, Amsterdam Netherlands; ^5^ Amsterdam UMC ‐ location VUmc, Department of Anatomy and Neurosciences, Neuroanatomy and Biobanking, Amsterdam Netherlands; ^6^ UNIL, 1015, Lausanne Switzerland

## Abstract

**Background:**

Neuropathological tau tangles in Alzerheimer’s disease (AD) have been roughly characterized into pre‐tangles, mature tangles and ghost tangles. However, to date, their maturation in the human brain is poorly understood. Here, we aimed to define the structure and composition of tangle maturation stages in the AD brain using correlative light and electron microscopy (CLEM).

**Method:**

Chemically fixed (4% paraformaldehyde, 0.1% glutaraldehyde) entorhinal and hippocampal formation of two diseased AD patients were analyzed using CLEM (6 more cases are currently being analyzed). Vibratome sections were stained with different relevant markers (AT8, AT180, 44744, etc; Amytracker; DAPI) and imaged using a fluorescence microscope. Brain sections were then processed for electron microscopy (heavy metal staining and resin embedding). Ultra‐thin alternate sections were collected on glass slides and electron microscopy grids.

**Result:**

We have characterized the ultrastructure of several mature tangles, dystrophic neurites, a few putative pre‐tangles and one putative ghost tangle. While we observed no tau fibrils in pre‐tangles, high contents of tau fibrils were present in mature tangles that often formed distinct clusters. Multilamellar bodies, damaged mitochondria and small membranous vesicles were present. Dystrophic neurites were swollen and filled with a mix of tau fibrils and multilamellar bodies. Often the twist of individual fibrils was visible. Fibrils in the putative ghost tangle had a fuzzy appearance, were densely packed and compartmentalized by a membrane. Compartmentalized clusters of multilamellar bodies were present as well.

**Conclusion:**

A prominent difference between mature tangles and neuritic pathology was the increased presence of multilamellar bodies in dystrophic neurites. This could be explained by differences in the aggregation process of tau fibrils, depending on the cellular context (neuron soma vs dendrite). In most previous studies, the staining with amyloid markers suggested the absence of fibrils in pre‐tangles, while one report found fibrils in pre‐tangles using EM. Our results support the hypothesis of the absence of tau fibrils in the earliest form of pre‐tangles. Further work is needed to identify the tau tangle stage at which fibrillar tau first occurs. The here presented preliminary data needs to be expanded with additional earlier and later stages of tau pathology.

